# Assessment of the Multifunctional Behavior of Lupin Peptide P7 and Its Metabolite Using an Integrated Strategy

**DOI:** 10.1021/acs.jafc.0c00130

**Published:** 2020-03-30

**Authors:** Carmen Lammi, Gilda Aiello, Luca Dellafiora, Carlotta Bollati, Giovanna Boschin, Giulia Ranaldi, Simonetta Ferruzza, Yula Sambuy, Gianni Galaverna, Anna Arnoldi

**Affiliations:** † Department of Pharmaceutical Sciences, University of Milan, 20133 Milan, Italy; ‡ Department of Food and Drug, University of Parma, 43124 Parma, Italy; § Food and Nutrition Research Centre, Council for Agricultural Research and Economics (CREA), 00178 Rome, Italy

**Keywords:** ACE, bioactive peptides, DPP-IV, hypotensive, intestinal transport, peptide

## Abstract

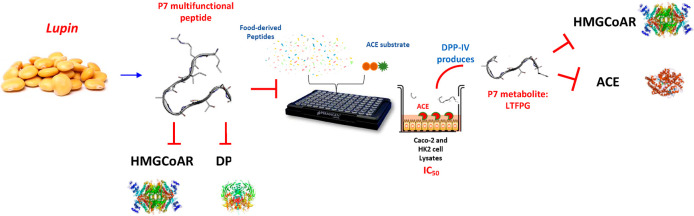

LTFPGSAED (P7) is a multifunctional hypocholesterolemic and hypoglycemic lupin peptide. While assessing its angiotensin-converting enzyme (ACE) inhibitory activity, it was more effective in intestinal Caco-2 cells (IC_50_ of 13.7 μM) than in renal HK-2 cells (IC_50_ of 79.6 μM). This discrepancy was explained by the metabolic transformation mediated by intestinal peptidases, which produced two main detected peptides, TFPGSAED and LTFPG. Indeed LTFPG, dynamically generated by intestinal dipeptidyl peptidase IV as well as its parent peptide P7 were linearly absorbed by mature Caco-2 cells. An *in silico* study demonstrated that the metabolite was a better ligand of the ACE enzyme than P7. These results are in agreement with an *in vivo* study, previously performed by Aluko et al., which has shown that LTFPG is an effective hypotensive peptide. Our work highlights the dynamic nature of bioactive food peptides that may be modulated by the metabolic activity of intestinal cells.

## Introduction

Recent literature indicates that some peptides obtained through the hydrolysis of different food proteins may provide favorable effects in the area of cardiovascular disease prevention, because they are characterized by hypocholesterolemic, hypoglycemic, or hypotensive activities.^1^ Among these peptides, those providing more than one activity are classified as multifunctional and are currently considered particularly interesting for practical applications.^2^

To express their activity *in vivo*, peptides masked within a protein sequence need not only be released by specific and selective proteases but may also be absorbed at the intestinal level and enter blood circulation to reach the target organs.^2^ Differentiated Caco-2 cells still represent the best available model system for intestinal transport studies.^3−5^ In fact, when these cells are differentiated on a permeable filter, they form a two-compartment system, where the apical (AP) compartment reproduces the intestinal lumen and the basolateral (BL) compartment reproduces the interstitial space.^6^ Recently, an evaluation of the transepithelial transport of a peptic lupin hydrolysate^7^ has shown that eight peptides are transported across the intestinal cells.^6^ One of these peptides was the nonapeptide LTFPGSAED, also named P7.^8^ Subsequent experiments have shown that P7 is a multifunctional peptide, because it is able to modulate cholesterol metabolism through inhibition of 3-hydroxy-3-methylglutaryl coenzyme A reductase (HMGCoAR)^8^ as well as to regulate glucose metabolism through dipeptidyl peptidase IV (DPP-IV) inhibition.^8−10^ Specifically, P7 reduces *in vitro* the activity of HMGCoAR in a dose–response manner and an IC_50_ of 68.4 μM. In human hepatic HepG2 cells, this inhibition leads to an upregulation of the low-density lipoprotein (LDL) receptor (LDLR) protein levels, through the activation of the sterol regulatory element-binding protein 2 (SREBP-2) pathway, and to an increase of LDL absorption from the extracellular environment, with a final hypocholesterolemic effect.^8^ In the area of diabetes prevention, P7 impairs the DPP-IV activity in different model systems: specifically, *in vitro* on the DPP-IV enzyme, where the IC_50_ was equal to 228 μM,^10^ in human intestinal Caco-2 cells with an IC_50_ of 223 μM, and on the circulating DPP-IV form in human serum with an activity reduction of 18.1 and 24.7% at the concentration of 100 and 300 μM, respectively.^9^

To further explore the potential multifunctional behavior of P7, the first objective of this work was an evaluation of its capacity to inhibit the activity of the angiotensin-converting enzyme (ACE, peptidyl dipeptidase A, EC 3.4.15.1), a key enzyme for blood pressure regulation. Therefore, a preliminary screening of the structures of P7 using BIOPEP (www.uwm.edu.pl/biochemia) had suggested that it might be compatible with a potential behavior as ACE inhibitors. Thus, lupin peptide activity as an ACE inhibitor was tested using two human cellular models, the former based on renal HK-2 cells, an immortalized proximal tubule epithelial cell line from normal adult human kidney, and the latter based on undifferentiated human intestinal Caco-2 cells, a reliable model of the enterocytes. Both cell systems are among those that mostly express ACE in the body. Even though the somatic ACE enzyme expressed by intestinal and renal cells do not seem to directly correlate with blood pressure regulation, it has the same sequence of the ACE expressed in the lung.

The fact that P7 was a more efficient ACE inhibitor in the Caco-2 cellular system than in the renal cellular system has suggested the hypothesis of a metabolic transformation of P7 in one or more active metabolites induced by Caco-2 cells, which are metabolically more active than renal cells. The second objective of the work was thus a study on the behavior of P7 in a differentiated Caco-2 cell model system aimed at investigating the intestinal cellular uptake as well as the possible concurrent degradation by active peptidases, expressed on the AP membranes, which may be accountable for the production of metabolites. After identification of an abundant metabolite, the third objective was to investigate its potential biological activities. In addition, a molecular modeling study was carried out to obtain a deeper comprehension of the interaction of P7 and its metabolite with the ACE structure. This *in silico* study was based on a structure-based modeling of both ACE domains (namely, the N and C domains) consisting of pharmacophoric analysis, docking simulations, rescoring procedures, and molecular dynamics.

## Materials and Methods

### Chemicals and Reagents

All reagents and solvents were purchased from commercial sources and used without further purification. For further details, see the Supporting Information.

### Cellular ACE Inhibitory Assays

HK-2 cells from the American Type Culture Collection (ATCC) were cultured using Dulbecco’s modified Eagle’s medium–F12 (DMEM–F12) containing 25 mM glucose, 4 mM stable l-glutamine, 100 units L^–1^ penicillin, and 100 μg L^–1^ streptomycin, supplemented with 10% heat-inactivated fetal bovine serum (FBS, Hyclone Laboratories, Logan, UT, U.S.A.). Caco-2 cells, obtained from Institut National de la Santé et de la Recherche Médicale (INSERM, Paris, France), were routinely subcultured at 50% density and maintained at 37 °C in a 90/10% air/CO_2_ atmosphere in DMEM containing 25 mM glucose, 3.7 g/L NaHCO_3_, 4 mM stable l-glutamine, 1% non-essential amino acids, 100 units/L penicillin, and 100 μg/L streptomycin (complete medium), supplemented with 10% heat-inactivated FBS.^11^

For the experiments, HK-2 and Caco-2 cells were seeded on 96-well plates at a density of 5 × 10^4^ cells/well for 24 h. The following day, cells were treated with 100 μL of P7 (0.1–250 μM) or vehicle in growth medium for 24 h at 37 °C. On the next day, cells were scraped in 30 μL of ice-cold ACE1 lysis buffer and transferred to an ice-cold microcentrifuge tube. After centrifugation at 13300*g* for 15 min at 4 °C, the supernatant was recovered and transferred to a new ice-cold tube. Total proteins were quantified by the Bradford method, and 2 μL of the supernatant (the equivalent of 2 μg of total proteins) was added to 18 μL of ACE1 lysis buffer in each well in a black 96-well plate with a clear bottom. For the background control, 20 μL of ACE1 lysis buffer was added to 20 μL of ACE1 assay buffer. Then, 20 μL of diluted ACE1 substrate [*o*-aminobenzoyl peptide (Abz-based peptide) substrate, 4% of ACE1 substrate in the assay buffer] was added in each well, except the background well, and the fluorescence (excitation/emission of 330/430 nm) was measured in a kinetic mode for 10 min at 37 °C.

### Caco-2 Cell Culture and Differentiation

For differentiation, Caco-2 cells were seeded on polycarbonate filters with a 12 mm diameter and 0.4 μm pore diameter (Transwell, Corning, Inc., Lowell, MA, U.S.A.) at a 3.5 × 10^5^ cells/cm^2^ density in complete medium supplemented with 10% FBS in both AP and BL compartments for 2 days to allow for the formation of a confluent cell monolayer. Starting from day 3 after seeding, cells were transferred to FBS-free medium in both compartments, supplemented with ITS [final concentration of 10 mg/L insulin (I), 5.5 mg/L transferrin (T), and 6.7 μg/L sodium selenite (S), GIBCO–Invitrogen, San Giuliano Milanese, Italy] only in the BL compartment, and allowed to differentiate for 18–21 days with regular medium changes 3 times weekly.^12^

### Cell Monolayer Integrity Evaluation

The transepithelial electrical resistance (TEER) of differentiated Caco-2 cells was measured at 37 °C using the voltmeter apparatus Millicell (Millipore Co., Billerica, MA, U.S.A.), immediately before and at the end of the transport experiments. In addition, at the end of transport experiments, cells were incubated from the AP side with 1 mM phenol red in phosphate-buffered saline (PBS) containing Ca^2+^ (0.9 mM) and Mg^2+^ (0.5 mM) for 1 h at 37 °C, to monitor the paracellular permeability of the cell monolayer. The BL solutions were then collected, and NaOH (70 μL, 0.1 N) was added before reading the absorbance at 560 nm by a microplate reader Synergy H1 from Biotek (Winooski, VT, U.S.A.). Phenol red passage was quantified using a standard phenol red curve. Only filters showing TEER values and phenol red passages similar to untreated control cells were considered for peptide transport analysis.

### Transepithelial Transport of P7 and LTFPG

Prior to experiments, the cell monolayer integrity and differentiation were checked by TEER measurement as described in detail above. Cells were then washed twice, and peptide transportation by intestinal cells was assayed. Transport experiments were performed in transport buffer solution (137 mM NaCl, 5.36 mM KCl, 1.26 mM CaCl_2_, 1.1 mM MgCl_2_, and 5.5 mM glucose) following conditions previously described.^13^ To reproduce the pH conditions existing *in vivo* in the small intestinal mucosa, AP solutions were maintained at pH 6.0 (buffered with 10 mM morpholinoethanesulfonic acid) and BL solutions were maintained at pH 7.4 (buffered with 10 mM *N*-2-hydroxyethylpiperazine-*N*-4-butanesulfonic acid). Prior to transport experiments, cells were washed twice with 500 μL of PBS containing Ca^2+^ and Mg^2+^. Peptide transportation by mature Caco-2 cells was assayed by loading the AP compartment with P7 and/or LTFPG (500 μM) in the AP transport solution (500 μL) and the BL compartment with the BL transport solution (700 μL). The plates were incubated at 37 °C, and the BL solutions were collected at different time points (i.e., 15, 30, 60, 90, and 120 min) and replaced with fresh solutions prewarmed at 37 °C. All BL solutions together with the AP solutions collected at the end of the transport experiment were stored at −80 °C prior to analysis. Three independent transport experiments were performed, each in duplicate.

### Targeted High-Performance Liquid Chromatography–Chip–Tandem Mass Spectrometry (HPLC–Chip–MS/MS) Analysis: Method Setup and Validation

Quantitative analysis of P7 in the AP and BL samples were carried out by ion trap mass spectrometry (MS) in multiple reaction monitoring (MRM) mode, monitoring two of the most intense diagnostic transitions, after optimization of the acquisition parameters, such as retention time, MS profile, and tandem mass spectrometry (MS/MS) fragmentation spectrum.^14,15^ All further details regarding liquid chromatography–tandem mass spectrometry (LC–MS/MS) operating conditions and method validations are described in the Supporting Information.

### Untargeted HPLC–Chip–MS/MS Analysis for the Detection of Metabolites

The metabolic degradation products deriving from the hydrolytic activity of brush border membrane peptidases were investigated by an untargeted approach (for further details, see the Supporting Information). Briefly, the extraction of MS/MS spectra for the metabolite analysis was conducted accepting a minimum sequence length of three amino acids and merging scans with the same precursor within a mass window of *m*/*z* ±0.4 in a time frame of ±5 s. Methionine oxidation, acetylation (K), pyroglutamic acid (N-termQ), and deamidated (N) were set as variable modifications; no enzyme was chosen as the digestive enzyme; and two missed cleavages were allowed. The MS/MS search was conducted against the subset of *Lupinus* protein sequences (8669 entries) downloaded from UNIProtKB (http://www.uniprot.org/). The mass tolerance of parent and fragments of the MS/MS data search was set at 1.0 Da for precursor ions and 0.8 Da for fragment ions, respectively. The auto-validation strategy in both peptide and protein polishing modes was performed using a false discovery rate (FDR) cutoff of ≤1.2%.

### Stability of P7 in the Presence of DPP-IV

The experiments were carried out in microcentrifuge tubes. Each reaction (100 μL) was prepared by adding the reagents in the following order: 1× DPP-IV assay buffer [20 mM Tris–HCl at pH 8.0 containing 100 mM NaCl and 1 mM ethylenediaminetetraacetic acid (EDTA)] (80 μL), P7 solution (10 μL, 500 μM), and finally DPP-IV (10 μL). Subsequently, the samples were mixed and kept at 37 °C in a thermoblock for 5, 30, and 120 min. At the end of the reactions, DPP-IV was inactivated by adding 200 μL of precooled acetonitrile (ACN) to each tube; then the samples were centrifuged for 10 min at 13300*g* at 4 °C; and the supernatant was collected. P7 and LTFPG were loaded onto the enrichment column (Zorbax 300SB-C18, 5 μm pore size) at a flow rate of 4 μL/min using isocratic 100% C solvent phase (99% water, 1% ACN, and 0.1% formic acid). After the cleanup, P7 and LTFPG were separated on a 150 mm × 75 μm analytical column (Zorbax300SB-C18, 5 μm pore size) at the constant flow rate of 300 nL/min. The LC solvent A was 95% water, 5% ACN, and 0.1% formic acid; solvent B was 5% water, 95% ACN, and 0.1% formic acid. The nanopump gradient program was as follows: 5% solvent B (0 min), 70% solvent B (0–8 min), and back to 5% solvent B in 2 min. Post-time was 10 min. The drying gas temperature was 300 °C, and the flow rate was 3 L/min (nitrogen). Data acquisition occurred in positive ionization mode. Capillary voltage was −1950 V, with an end plate offset of −500 V. Mass spectra were acquired under MRM conditions by monitoring *m*/*z* 469.8 and 534.2 for P7 and LTFPG, respectively.

### *In Vitro* DPP-IV Inhibitory Activity Assay

The experiments were carried out in a half-volume 96-well solid plate (white) with LTFPG at the final concentrations of 10, 100, and 500 μM and using conditions previously optimized.^10^ For further details, see the Supporting Information

### *In Vitro* Assessment of the HMGCoAR Inhibitory Activity

The assay buffer, NADPH, substrate solution, and HMGCoAR were provided in the HMGCoAR assay kit (Sigma). The experiments were carried out testing LTFPG at 100 and 250 μM at 37 °C in agreement with the conditions previously reported.^8^ For further details, see the Supporting Information.

### *In Silico* Study

The molecular modeling study aimed at describing the interaction of peptides with both the N and C domains of human ACE. The study relied on pharmacophore modeling, docking studies, and molecular dynamic (MD) simulations, as detailed below.

#### Model Preparation

The models for the C and N domains of human ACE were derived from the three-dimensional structures recorded into the Protein Data Bank (http://www.rcsb.org) with PDB codes 4APH and 4BZS, respectively.^16,17^ Protein structures were processed using the software Sybyl, version 8.1 (www.certara.com), as previously reported.^18^ Briefly, all atoms of both structures were checked for atom- and bond-type assignments, and amino- and carboxyl-terminal groups were set as protonated and deprotonated, respectively. Hydrogen atoms were computationally added to the protein and energetically minimized using the Powell algorithm (the coverage gradient was set at ≤0.5 kcal mol^–1^ Å^–1^ with a maximum of 1500 cycles). All sets of small molecules but not the Zn ions, co-crystallized within the catalytic sites, were removed to prepare the model for docking simulations. Peptides were designed using the “Build Protein” tool of the “Biopolymer” module of Sybyl, version 8.1 (www.certara.com). Then, they were energetically minimized using the Powell algorithm with a coverage gradient of ≤0.05 kcal mol^–1^ Å^–1^ and a maximum of 500 cycles.

#### Pharmacophoric Modeling

The pharmacophoric modeling aimed at describing the physicochemical properties of catalytic sites in terms of distribution of hydrophobic and hydrophilic features. The binding site of both domains of ACE was defined using the Flapsite tool of the FLAP software, while the GRID algorithm was used to investigate the corresponding pharmacophoric space.^19,20^ In particular, the DRY probe was used to describe potential hydrophobic interactions, while the sp^2^ carbonyl oxygen (O) and neutral flat amino (N1) probes were used to describe the hydrogen bond acceptor and donor capacities of the target, respectively.

#### Docking Study and Rescoring Procedure

The docking study aimed at investigating the architectures of peptide binding within the catalytic sites of ACE domains. The GOLD software (version 5.7)^21^ was used to perform all of the docking simulations, while a rescoring procedure using the HINT scoring function^22^ was performed for the better evaluation of the peptide–ACE interaction. In particular, HINT score relates to the free energy of binding (the higher the score means the stronger the interaction, while negative scores indicate the lack of appreciable interaction).^23^ Notably, the coupling of docking simulations using GOLD and rescoring procedures using HINT already succeed in identifying enzyme inhibitors as previously shown.^23−25^ Software setting and docking protocol were used as reported previously.^25^ Briefly, the explorable space available for docking peptides was set at 10 Å around the Zn ion. In addition, the interaction of the C-terminal carboxylic group of peptides was restrained in agreement with the arrangement of the carboxylic group of captopril, as reported by a crystallographic study,^26^ to speed up the spatial search.

GOLD uses a Lamarckian genetic algorithm, and scores may slightly change from run to run. Therefore, to exclude a non-causative score assignment, simulations were run in quintuplicate and the mean values are reported, in agreement with previous studies.^25^

#### MD Simulations

MD simulations were performed to study the dynamic of interactions between peptides and the ACE domains over time. The best scored binding poses calculated by docking simulations were used as input for MD. MD simulations were performed using GROMACS (version 5.1.4) with CHARMM27 all-atom force field parameter support,^27^ in agreement with a previous study.^28^ Briefly, protein–peptide complexes solvated with SPCE waters in a cubic periodic boundary condition, and counterions (Na^+^ and Cl^–^) were added to neutralize the system. Prior to MD simulation, the systems were energetically minimized to avoid steric clashes and to correct improper geometries using the steepest descent algorithm with a maximum of 5000 steps. Afterward, all of the systems underwent isothermal (300 K, coupling time of 2 ps) and isobaric (1 bar, coupling time of 2 ps) 100 ps simulations before running 50 ns simulations (300 K with a coupling time of 0.1 ps and 1 bar with a coupling time of 2.0 ps).

### Statistical Analysis

All liquid chromatography–mass spectrometry (LC–MS) analyses were run in triplicate on each biological replicate. Statistical analysis, including determination of linear regression, average, standard deviation (sd), and coefficient of variance (CV), was performed using GraphPad Prism 7 (GraphPad Software, La Jolla, CA, U.S.A.). Values were expressed as the mean ± sd. For the experiments aimed at evaluating the bioactivity of P7 and LTFPG, statistical analyses were carried out by one-way analysis of variance (ANOVA) (GraphPad Prism 7), followed by Brown–Forsythe’s test. Values were expressed as the mean ± sd. *p* values of <0.05 were considered to be significant.

## Results

### P7 Inhibits the ACE Activity Expressed by Human Renal Cells and Intestinal Caco-2 Cells in Different Ways

**Figure 1:**
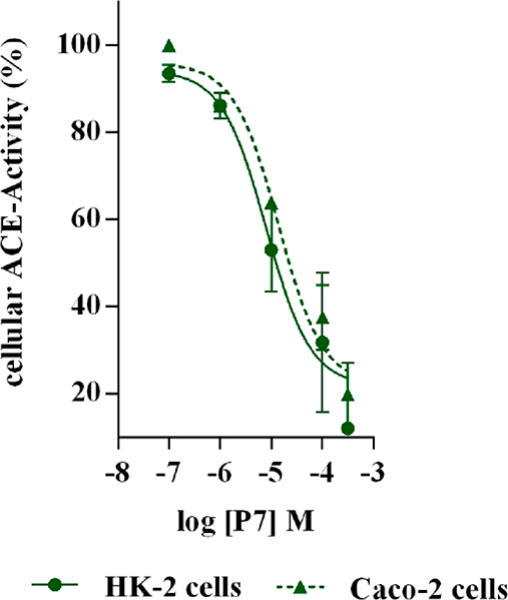
Evaluation of the ACE inhibitory activity of P7 in renal HK-2 and intestinal Caco-2 cells. P7 reduces the ACE activity with a dose–response trend and IC_50_ of 79.6 ± 0.20 and 13.7 ± 0.28 μM, respectively. Data represent the mean ± sd of three independent experiments performed in triplicate.

To obtain a deeper characterization of the multifunctional behavior of P7, its ACE inhibitory activity was investigated using a cell-based assay recently optimized in our laboratory, which is based on human renal HK-2 and intestinal Caco-2 cells.^29^ After treatment of both cell systems with P7, the ACE activity was measured directly in the cell lysates using a fluorescent ACE substrate: in this assay, the fluorescent signal is proportional to the enzyme activity. As shown in Figure 1, P7 inhibited the enzyme activity in both renal HK-2 and Caco-2 cell systems with a dose–response trend and IC_50_ values equal to 79.6 ± 0.20 and 13.7 ± 0.28 μM, respectively; i.e., P7 is 6-fold more active at the intestinal level than the renal level.

The differences between the results in the two cellular systems may be possibly explained considering the propensity of P7 to undergo a metabolic degradation by active peptidases. In fact, the different metabolic patterns of each cell line may be responsible for the generation of one or more breakdown fragments each endowed with specific activities that may be different from those of the parent peptide. It was thus hypothesized that the metabolic activities expressed by the Caco-2 cells and, in particular, the hydrolytic activity of brush border peptidases might actively influence the behavior of P7 through the production of smaller metabolic fragments, which might be more active than the parent peptide. Therefore, the subsequent in-depth experimentation was aimed at obtaining a solid explanation of this phenomenon by evaluating the behavior of P7 in the presence of mature intestinal Caco-2 cells.

### Transport and Metabolism of P7 Alone or in a Mixture with Other Peptides across Caco-2 Cells

The following experiments were dedicated evaluating the transport and metabolism of P7 in differentiated Caco-2 cell monolayers. From a dynamic point of view, the transport process of bioactive peptides may be different when they are present in a complex hydrolysate or when they are alone. For this reason, it was decided to investigate the kinetics of P7 transport in two different conditions, i.e., when P7 was alone or when it was in a mixture. The mixture was prepared by mixing P7 with two other lupin peptides, namely, YDFYPSSTKDQQS (P3) and LILPKHSDAD (P5), which had already been demonstrated to be transported in the same system. Each peptide was tested at the concentration of 500 μM in the AP compartment.

**Figure 2:**
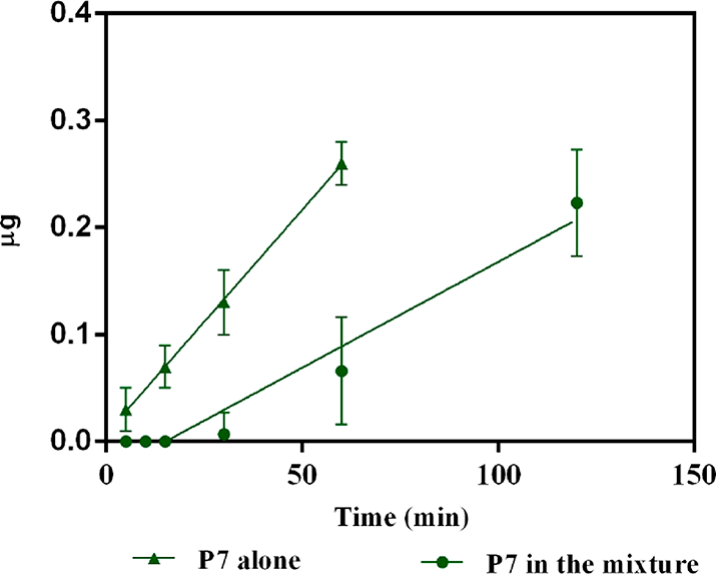
Transport of P7 across Caco-2 cells. Quantification of P7 in the BL compartment as a function of time: trend of P7 alone (green triangle) and in a mixture (green dot). Data represent the mean ± sd of three independent experiments performed in triplicate.

As shown in Figure 2, in both systems, P7 was linearly absorbed across the Caco-2 cell monolayer as a function of time. In the case of P7 alone, the rate of transport was 4.2 ± 0.6 ng mL^–1^ min^–1^ (*R*^2^ = 0.999), with a lag period for transport of 0.5 min, whereas in the mixture, the rate was 1.98 ± 0.21 ng mL^–1^ min^–1^ (*R*^2^ = 0.955), with a lag period of 27.7 min (Figure 2). The much slower rate observed when P7 was in a mixture suggests that peptide–peptide or peptide–peptidase interactions actively modulate the dynamic of its transport. In fact, the presence of other peptides may preferentially favor a certain transport selectivity. Moreover, after 60 min of incubation, the amount of P7 in the BL compartment was about 4-fold higher (0.26 ± 0.02 μg, equal to 0.278 nmol) when it was tested alone than when it was tested in the mixture (0.06 ± 0.003 μg, equal to 0.064 nmol). Moreover, in the latter conditions, 2 h were required to reach about the same absorbed amount (0.22 ± 0.5 μg, 0.235 nmol). In all cases, the incubation with the peptides did not affect the monolayer integrity as monitored by TEER values and phenol red passage (data not shown), thus indicating that the passage was transcellular rather than paracellular.

### Analysis of the Metabolites Produced by Caco-2 Cells

In their AP sides, mature enterocytes develop functional structures, the microvilli, on whose surface many active carriers and metabolic enzymes are expressed. In the same way, differentiated Caco-2 cells express in their AP membrane a wide range of peptidases, including also DPP-IV and ACE. From a physiological point of view, the dynamic equilibrium between bioactive peptide transport and degradation is crucially important. Therefore, under the hypothesis that the low transport rate observed for P7 might be attributed to competing *in situ* degradation by the hydrolytic activity of brush border peptidases, the AP solutions, collected after 120 min of the transport experiment, were analyzed looking for metabolic degradation products. Two peptides, namely, TFPGSAED (with *m*/*z* 823.20) and LTFPG (with *m*/*z* 534.29), were identified deriving from the loss of the first amino acid (L) from the N-terminal side and the loss of the last four amino acid residues (SAED) from the C-terminal side, respectively (Table 1). These results suggest that P7 is a substrate of two different peptidases: leucine aminopeptidase (LAP) catalyzes the hydrolysis of the leucine residues at the N terminus of P7, generating the TFPGSAED, while among all of the intestinal endopeptidases, DPP-IV might be responsible for the formation of the LTFPG fragment. The fact that both metabolites are detected only in the AP samples of the experiments when P7 is tested in the mixture underlines different kinetics in the generation of breakdown fragments. Possibly, the presence of other peptides in the AP compartment may protect the two major metabolites from further degradation by the intestinal peptidase that expresses their activities on different substrates. The fact that, when it is tested in a mixture, P7 can be detected in the BL medium only after 27 min suggests that, over that period of time, the degradation into the two metabolites prevails over transport. Possibly, protected by the presence of the other peptides against degradation, the metabolites might impair the P7 transport, thus delaying its passage and detection in the BL medium. However, the confirmation of this hypothesis would require further studies. Conversely, when P7 is individually tested, it is rapidly absorbed, without lag period, and its major metabolites are not detectable in the AP medium at the end of the experiment (60 min), possibly as a result of their total degradation by intestinal peptidases, generating smaller breakdown fragments that are intrinsically difficulty to assign, such as tri- and dipeptides. Alternatively, the cited metabolites might have been produced but remain below the detection limit.

**Table 1:** Metabolites Produced at the AP Side When P7 Is Tested as Individual Species and within a Mixture

peptide sequence	[M + H]^+^	*m*/*z*	mixture	alone
LTFPGSAED (P7, parent peptide)^a^	936.43	469.80	×	×
TFPGSAED (AP metabolite)	823.20	823.20	×	
LTFPG (AP metabolite)^a^	534.29	534.29	×	

a P7 and LTFPG were test at 1 mg/mL, respectively.

### LTFPG Is a Metabolic Product of the Intestinal DPP-IV Activity

**Figure 3:**
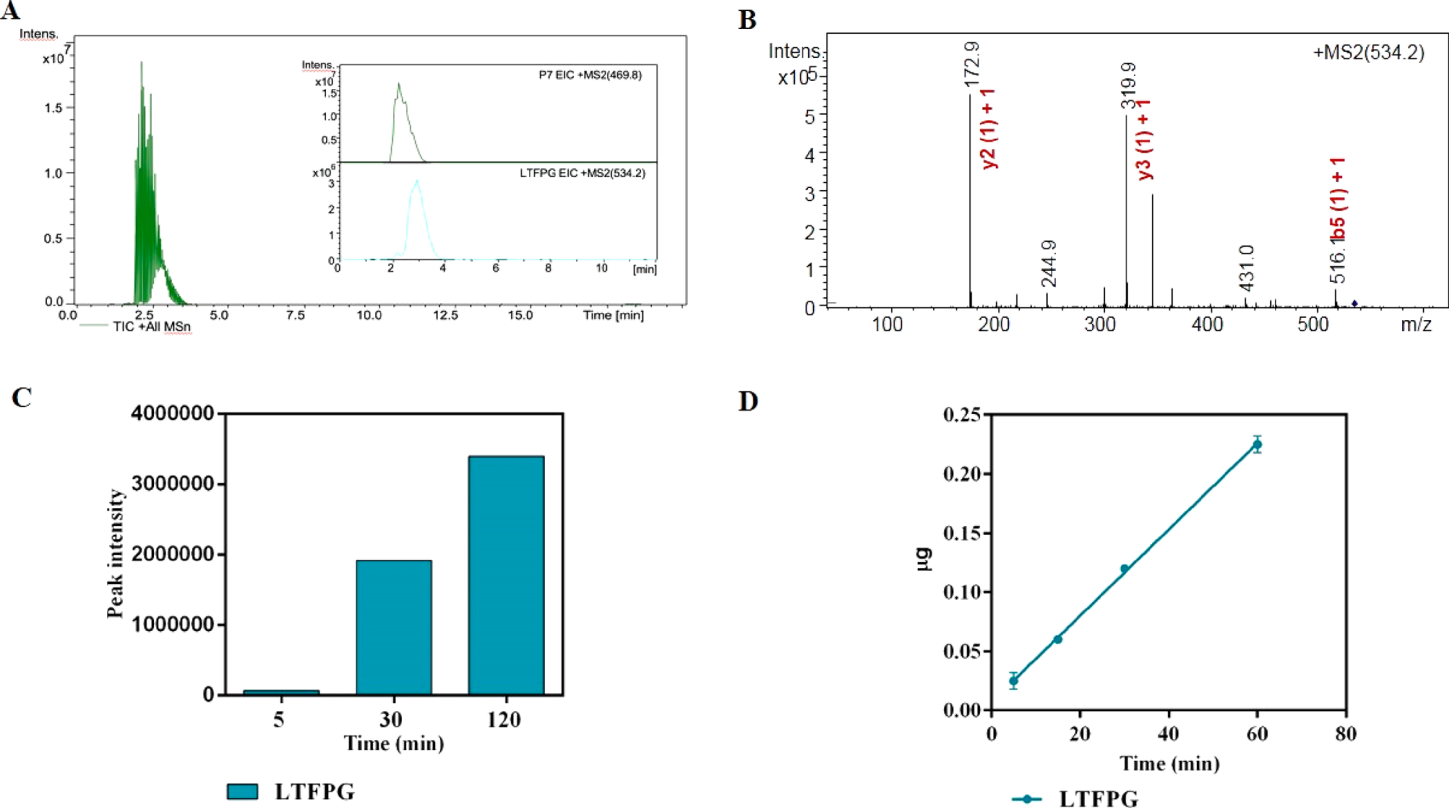
Incubation of LTFPG with DPP-IV and transport in Caco-2 monolayers. Incubation with DPP-IV: (A) TIC and EIC chromatograms of P7 and LTFPG, respectively, (B) MS/MS spectrum of LTFPG, and (C) peak intensity of LTFPG as a function of time of incubation with DPP-IV. Transport experiments in Caco-2 monolayers: (D) linear transport of LTFPG as a function of time.

The following investigations were focused on LTFPG, because TFPGSAED is very similar to the parent peptide P7. To verify whether DPP-IV was responsible for the production of LTFPG from P7, an *in vitro* biochemical test was performed using the purified recombinant enzyme. P7 (500 μM) was incubated with DPP-IV for 5, 30, and 120 min, and the formation of LTFPG was monitored by LC–MS. LTFPG was clearly detectable after 2 h of incubation, as indicated by Figure 3A that reports the total ion current (TIC) and extracted ion current (EIC) chromatograms of P7 and LTFPG. The MS/MS spectra of LTFPG are shown in Figure 3B. As indicated by Figure 3C, the peak area of LTFPG increases as a function of the incubation time.

In addition, a transport experiment was performed using mature Caco-2 cells (Figure 3D): the rate of transport of LTFPG alone (incubated in the AP compartment at the concentration of 500 μM) was equal to 3.7 ± 0.8 ng mL^–1^ min^–1^ (*R*^2^ = 0.997) without a lag period. Interestingly, after 60 min of transport, the concentration of LTFPG in the BL compartment (0.22 ± 0.003 μg, equal to 0.412 nmol) was much higher than that of the parent peptide P7 tested alone (0.26 ± 0.02 μg, equal to 0.278 nmol). This result suggests that LTFPG is either efficiently transported or poorly metabolized by intestinal Caco-2 cells. Additional experiments showed that LTFPG is transported also in the presence of wortmannin, a well-known inhibitor of the transcellular passage (see Figure 1S of the Supporting Information), suggesting that the mechanism of transport may involve the paracellular route. It is important to underline, however, that dedicated experiments would be required for a complete characterization of the LTFPG transport mechanism.

### *In Silico* Studies of the ACE Inhibitory Properties of P7 and Its Metabolite

Recent literature indicates that indeed LTFPG is a hypotensive peptide. In particular, Aluko and co-workers have identified this peptide after the hydrolysis of pea seed provicilin with thermolysin^30^ and have demonstrated that it has moderate but significant *in vitro* inhibitory activities on ACE and renin. Moreover, when orally administered to spontaneously hypertensive rats (SHRs) at a dose of 30 mg/kg of body weight, LTFPG produces a fast and efficient decrease in systolic blood pressure with a maximum of −37 mmHg after 2 h. These results demonstrate a hypotensive activity

On the basis of these considerations, an *in silico* study was carried out to compare the mechanisms through which P7 and LTFPG interact with the ACE enzyme, using a molecular modeling approach, in agreement with a previous study.^25^ Briefly, an integrated use of docking simulations, rescoring procedures, pharmacophoric analysis, and MD simulations were used to estimate the capacity of peptides to favorably and stably interact with the two catalytic sites of the enzyme.

In more detail, docking simulations provided the binding poses of the peptide, which were rescored using the HINT scoring function to find the most likely and favored one. The coupled use of docking simulations and HINT as a rescoring function was chosen, because it previously succeeded to estimate the favors of peptide–enzyme complex formation.^25,31^ In particular, the HINT score may correlate to the favors of binding, as previously reported (the higher the score, the more favored the expected interaction).^23^

P7 showed negative HINT scores within both sites (−932 and −1810 units within the N and C domains, respectively), suggesting a low fitting within the two catalytic sites of ACE. This evidence was in line with its moderate *in vitro* ACE inhibitory activity (10.9 ± 0.95% at 1.0 mg/mL), as mentioned above (see Table 1S of the Supporting Information). Therefore, P7 was not investigated further in the computational assessment.

**Figure 4:**
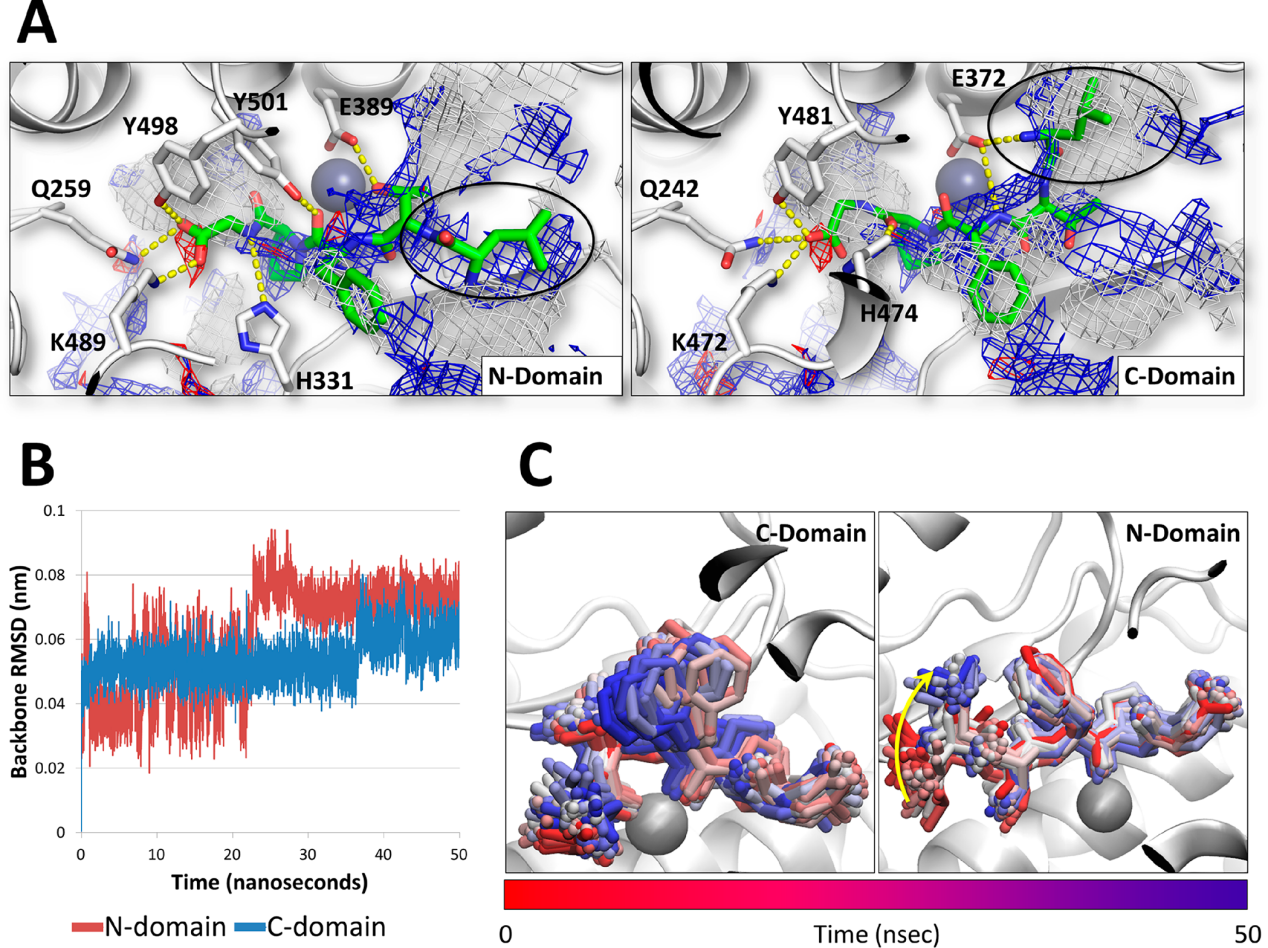
Computational results of LTFPG. (A) Binding poses of LTFPG within the ACE catalytic sites and respective pharmacophoric analysis. The protein is represented in a cartoon, while peptides and residues involved in polar interactions are represented in sticks. Zn ions are represented by spheres. Gray, red, and blue meshes indicate regions sterically and energetically favorable to receive hydrophobic, hydrogen bond acceptor, and hydrogen bond donor groups, respectively. Polar interactions are indicated by yellow dotted lines. The circles indicate the N terminus of the peptide. (B) RMSD analysis of LTFPG within the two catalytic sites of ACE. (C) Time-step representation of LPYP trajectories within the N and C domains of ACE. The red to blue color switch indicates the stepwise changes of ligand coordinates over time (50 ns). The yellow arrow indicates the movement of the N terminus of LTFPG along the simulation.

Conversely, LTFPG showed relatively high and positive scores in both catalytic sites (975 and 426 HINT score units within the N- and C-terminal domains, respectively), suggesting a theoretical fitting higher than that of P7. This result is in accordance to the higher activity of LTFPG with respect to the parent peptide P7, and it clearly points to the higher capability of the former to better satisfy the physicochemical requirements of ACE catalytic sites. The analysis of the poses revealed that LTFPG had a very similar architecture of binding in both sites, with the exception of a slightly different arrangement of its N-terminal residues among the two. This result may explain the diverse scores observed in the two sites. The analysis of MD results showed a slightly different behavior of LTFPG between the two catalytic sites (Figure 4B). In particular, the root-mean-square deviation (RMSD) analysis was used to monitor the geometrical stability within the two catalytic sites over time, in agreement with a previous study. The results collected showed stable interaction of LTFPG within the catalytic site of the N terminal from the half of simulation because it showed a steady geometry from 25 ns until the end of the simulation. Conversely, within the C domain, LTFPG showed a discrete increase of RMSD in the last part of the simulation, although showing a steady geometry until the end of simulation. In addition, the analysis of LTFPG trajectories showed its capability to persist within the two catalytic sites over time. Specifically, concerning the interaction with the catalytic site of the ACE N domain, the reorganization of the N terminus of LTFPG along the simulation explained the high RMSD values observed in the first part of the simulation. Overall, the results collected pointed to the capability of LTFPG to interact and stably persist within both the catalytic sites of ACE.

### Evaluation of the Inhibitory Activity of LTFPG on DPP-IV and HMGCoAR

**Figure 5:**
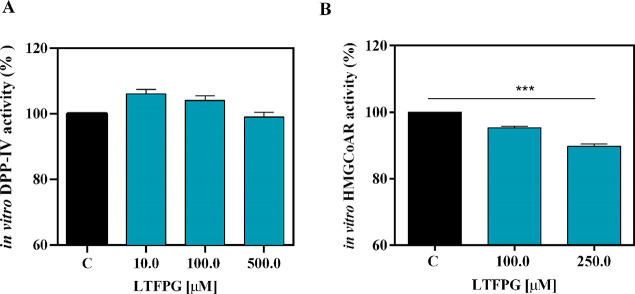
Investigation of LTFPG biological activities. Effects of LTFPG on the *in vitro* (A) DPP-IV and (B) HMGCoAR activities. Data represent the mean ± sd of three independent experiments performed in triplicate. C = control sample. (∗∗∗) *p* < 0.001.

It was decided to verify whether LTFPG retained the multifunctional activities of the parent compound P7. The results of these experiments showed that LTFPG loses the ability to reduce the *in vitro* activity of DPP-IV (Figure 5A), whereas it maintains a modest ability to reduce the *in vitro* HMGCoAR activity. In fact, it inhibits the enzyme by 4.7 ± 0.3 and 10.3 ± 0.8% at 100 and 250 μM Figure 5B).

## Discussion

Although there is an increasing number of papers that underline the interesting biological properties of food peptides, the issues of their metabolism and transport still remain relevant issues of discussion. In particular, these phenomena have been invocated to explain the discrepancy observed between *in vitro* assays and *in vivo* results. For example, there are many reports in the literature on the ACE inhibitory activity of different food-derived peptides.^32^ In all of these studies, the biochemical characterization is carried out using tests on the purified recombinant ACE enzymes from lung or kidney of different animal species, such as pig and rabbit. These biochemical tools, involving a purified ACE enzyme and a standard substrate, provide only an incomplete characterization of the activity and represent a rudimental way of screening, which does not always correlate with the hypotensive effect observed in experimental studies that are usually performed using SHRs as the model system. For example, IQW and LKP are two peptides derived from a thermolysin–pepsin ovotransferrin hydrolysate. IQW seems the better ACE inhibitor in the biochemical test, having an IC_50_ value equal to 1.56 μM versus 2.93 μM of LKP, but when they are tested *in vivo* in the SHR model, IQW is the less effective, because it induces a −21.0 mmHg decrease of the BP, whereas LKP induces a −30.0 mmHg decrease.^33,34^ Recently, three peptides, WYT, SVYT, and IPAGV, identified in a hempseed hydrolysate, have been shown to exert an *in vitro* ACE inhibitory activity of 89.0, 79.0, and 60.0% at 0.5 mg/mL, respectively. However, IPAGV, the least active *in vitro*, was the most active in reducing the BP of SHR (−40.0 mmHg).^35^ Moreover, FKGRYYP, LKP, and IKW, three peptides identified from meat-derived hydrolysates obtained using thermolysin, are totally ineffective *in vivo* on SHRs, although they reduce *in vitro* the ACE activity, with IC_50_ values equal to 0.55, 0.32, and 0.21 μM, respectively.^36^

In addition, more and more works underline the possibility that, in some cases, metabolism may generate a fragment whose activity is enhanced and/or shifted to different targets. This is the case of peptide P7 that in itself is a poor inhibitor of the ACE activity (as also shown here by *in silico* outcomes), whereas the metabolic transformation induced by DPP-IV produced the active peptide LTFPG, whose hypotensive activity has been demonstrated either *in vitro* or *in vivo* in the SHR model.^30^

The DPP-IV ability to generate the active LTFPG fragment, after P7 degradation, highlights an additional aspect of the previously described DPP-IV inhibitory nature of P7.^9,10^ In general, three modes are used to describe the nature of enzyme inhibition: true, substrate, and prodrug type.^37^ True inhibitors are not degraded during incubation with the enzyme, whereas substrate and prodrug inhibitors are metabolized by the enzyme. DPP-IV inhibitors are classified on the basis of their stability to the hydrolytic action of DPP-IV *per se*.^38^ In this context, our results clearly confirm that P7 is a substrate of DPP-IV that acts as a competitive inhibitor, because it is subjected to DPP-IV hydrolysis. P7, however, is not a DPP-IV prodrug inhibitor, because LTFPG loses the ability to reduce the *in vitro* DPP-IV enzyme activity (Figure 5A). Recently, a molecular docking study has investigated the P7 interaction within the catalytic site of the DPP-IV enzyme.^10^ Results suggest that the P7 C terminal interacts with Arg358 and Arg356 and is engaged in an extended ionic network, also involving the side chain of the C-terminal residue (Asp9 in P7) and Arg429. Moreover, the contacts stabilized by the N terminal elicit the already described ion pairs with Glu205 and Glu206 in the active peptide P7. Finally, P7 includes an aromatic residue (Phe3) engaged in a rich set of π–π stacking involving Tyr547, Trp629, and His740.^10^ Thus, it is clear that, even though the P7 metabolite may potentially interact through the N-terminal residues with the catalytic side of the enzyme, this interaction is not further stabilized by the C terminal of the peptide, explaining why LTFPG does not act as a DPP-IV prodrug inhibitor.

Differently, LTFPG maintains a modest ability to reduce *in vitro* the HMGCoAR activity (by 4.7 ± 0.3 and 10.3 ± 0.8% at 100 and 250 μM, respectively); however, it is much less potent than the parent peptide P7 (IC_50_ of 68.4 μM).^8^ To function as a competitive inhibitor of HMGCoAR, a peptide should mimic the hydroxymethylglutaryl moiety. To achieve this goal, the conformation and side chain groups play a more important role than the total hydrophobicity. Moreover, the correlation of the inhibitory activity with the peptide length has not yet been established, while it has been confirmed that a Leu, Ile, and/or Tyr residue at the N terminal and a Glu residue at the C terminal play important roles for the peptide inhibitory property.^39,40^ In fact, the *in silico* prediction of the P7 binding mode within the catalytic site of the enzyme suggests that the P7-HMGCoAR complex may be stabilized by a set of interactions, which can be subdivided into three groups: (1) The positively charged N terminal elicits ion pairs with Glu559 and Asp767, reinforced by hydrogen bonds with surrounding Thr557 and Thr558. (2) Negatively charged residues located at the C-terminal tail (including the C terminal itself) are engaged in ionic contacts with Arg568, Arg571, and Lys722. (3) Hydrophobic residues located at the N terminal are involved in hydrophobic interactions with apolar residues (e.g., Leu76, Ile536, Leu562, Met655, and Met657).^6^ Thus, it appears that, as a result of the hydrolytic activity of DPP-IV, LTFPG maintains the set of interactions that involve the N-terminal side of the peptide but drastically loses the important set of interactions between the C-terminal side of the peptide and Arg568, Arg571, and Lys722, which stabilize the complex peptide catalytic site of the enzyme, with a consequent reduction of LTFPG inhibitory potency.

In conclusion, the couple P7 and LTFPG represent an exemplary case of the multiple facets of the behavior of some multifunctional peptides: the degradation of P7, which is hypocholesterolemic and hypoglycemic, produces a metabolite that loses these activities but becomes hypotensive. This study underlines how, in the field of multifunctional peptides, the overall activities may be attributed to the concomitant presence of metabolites with the same or also new bioactivity. This new vision highlights the dynamic nature of bioactive food peptides that may be modulated by the metabolic activity of intestinal cells. This aspect is still mostly underestimated, because the identification and characterization of multifunctional peptides from food proteins is still addressed using traditional and static approaches.

## Supporting Information Available

Information of chemicals and reagents used in this study and technical details of *in vitro* experiments and HPLC–Chip–MS/MS analysis (PDF)

## Abbreviations Used

ACE: angiotensin-converting enzyme
ACN: acetonitrile
AP: apical
BL: basolateral
CV: coefficient of variance
DMEM: Dulbecco’s modified Eagle’s medium
DPP-IV: dipeptidyl peptidase IV
EIC: extracted ion current
FBS: fetal bovine serum
HMGCoAR: 3-hydroxy-3-methylglutaryl coenzyme A reductase
ITS: insulin–transferrin–selenium
LAP: leucine aminopeptidase
LC–MS/MS: liquid chromatography–tandem mass spectrometry
LDL: low-density lipoprotein
LDLR: low-density lipoprotein receptor
MD: molecular dynamic
MRM: multiple reaction monitoring
MS/MS: tandem mass spectrometry
NADPH: nicotinamide adenine dinucleotide phosphate
PBS: phosphate-buffered saline
PDB: Protein Data Bank
RMSD: root-mean-square deviation
sd: standard deviation
SHR: spontaneously hypertensive rat
SREBP-2: sterol regulatory element-binding protein 2
TEER: transepithelial electrical resistance
TIC: total ion current
