# Supplementary material for: Assessment of the Multifunctional Behavior of Lupin Peptide P7 and Its Metabolite Using an Integrated Strategy
Source: J Agric Food Chem. 2020 Mar 30;68(46):13179–88. doi: 10.1021/acs.jafc.0c00130 (PMC7997369; doi:10.1021/acs.jafc.0c00130)
Supplement: Supplementary file 1 — jf0c00130_si_001.pdf [file jf0c00130_si_001.pdf]

Assessment of multifunctional behavior of lupin P7 and its metabolite using an integrate strategy

Lammi Carmen[1]*, Aiello Gilda[1], Dellafiora Luca[2], Bollati Carlotta[1], Boschin Giovanna[1], Ranaldi Giulia[3], Ferruzza Simonetta[3], Sambuy Yula[3], Galaverna Gianni[2], Arnoldi Anna[1]

1. Department of Pharmaceutical Sciences, University of Milan, Milan, Italy

2. Department of Food and Drug, University of Parma, Parma, Italy.

3. CREA, Food and Nutrition Research Centre, Rome, Italy

*Corresponding author: Phone: +39-0250319912; FAX: +39-0250319372; Email: carmen.lammi@unimi.it

Materials and methods

Chemicals and reagents

YDFYPSSTKDQQS (**P3**), LILPKHSDAD (**P5**), LTFPGSAED (**P7**), and LTFPG were synthesized (> 95% purity by HPLC) by GenScript (Piscataway, New Jersey, USA). Dulbecco's Modified Eagle Medium (DMEM) was from GIBCO (Thermo Fisher Scientific, Waltham, MA USA). Fetal Bovine Serum (FBS) was from Hyclone Laboratories (Logan, UT, USA). Stable L-glutamine, 1% non-essential amino acids, and penicillin/streptomycin were from Euroclone (Milan, Italy). Formic acid and acetonitrile (ACN) were from Sigma-Aldrich (Milan, Italy). LC-grade H_2O (18 MΩcm) was prepared with a Milli-QH_2O purification system (Millipore, Bedford, MA, USA). LC-grade H_2O (18 MΩcm) was prepared with a Milli-QH_2O purification system (Millipore, Bedford, MA, USA). Centrifugal filter devices (cutoff 3 kDa) were from Amicon Bioseparations (Millipore Corporation, Bedford, MA, USA). ACE1 Activity Assay Kit (Biovision, Milpitas Blvd., Milpitias, CA, USA)

Assessment of the in vitro HMGCoAR activity

The assay buffer, NADPH, substrate solution, and HMGCoAR were provided in the HMGCoAR Assay Kit (Sigma). The experiments were carried out following the manufacturer's instructions at 37 °C. In particular, each reaction (200 μL) was prepared adding the reagents in the following order: 1 X assay buffer, LTFPG (100.0 and 250.0 μM) or vehicle (C), the NADPH (4 μL), the substrate solution (12 μL), and finally the HMGCoAR (catalytic domain) (2 μL). Subsequently, the samples were mixed and the absorbance at 340 nm read by a microplate reader Synergy H1 from Biotek at time 0 and 10 min. The HMGCoAR-dependent oxidation of NADPH and the inhibition properties of lupin peptides were measured by absorbance reduction, which is directly proportional to enzyme activity.

In vitro ACE-inhibitory assay

Peptide P7 were tested evaluating hippuric acid (HA) formation from hippuryl-histidyl-leucine (HHL), as mimic substrate for angiotensin I. 100 μL of 2.5 mM HHL in 100 mM tris-HCOOH, 300 mM NaCl pH 8.3 (buffer 1) was mixed with 30 μL of peptide solution in buffer 1. Six different concentrations of sample were tested and each solution was tested twice. Samples were preincubated at 37 °C for 15 min, then 15 μL of ACE solution, in 100 mM tris-HCOOH, 300 nM NaCl, 10 μM ZnCl2, pH 8.3, were added. Samples were incubated at 37 °C, and after 60 min 125 μL of 0.1 M HCl were added. The aqueous solution was extracted twice with 600 μL of ethyl acetate; the solvent was evaporated, then the residue was dissolved in 500 μL of buffer 1. HPLC analyses were performed with a HPLC 1200 Series (Agilent Technologies, Santa Clara, US) equipped with an autosampler using and a column Lichrospher 100 C18 (4.6 × 250 mm, 5 μm; Grace, Italy). The analytical parameters were: flow rate, 0.5 mL/min; detector, λ 228 nm; mobile phase, water and MeCN, gradient elution from 5 to 60% MeCN in 10min and 60% MeCN for 2 min, then back to 5% MeCN in 3 min; injection volume, 10 μL; Rt (HA), 4.2 min.

The evaluation of the inhibition of ACE activity was based on the comparison between the concentrations of HA in the presence or absence of the inhibitor (Inhibitor Blank). The phenomenon of autolysis of HHL to give HA was evaluated by a reaction blank, i.e. a sample with the higher

inhibitor concentration but without the enzyme. The percentage of ACE inhibition was calculated considering the area of the HA peak with the following formula:

$$\% \text{ ACE INHIBITION} = \left[\frac{(A_{IB}-A_{N})}{(A_{IB}-A_{RB})} \right] * 100$$

where AIB is the area of HA in the Inhibitor Blank (IB) sample (i.e., sample with enzyme but without inhibitor), AN is the area of HA in the samples containing different inhibitor amounts, and ARB is the area of HA in the Reaction Blank (RB) sample (i.e., sample without enzyme and with inhibitor at the highest concentration). The percentages of ACE inhibition were plotted vs Log10 inhibitor concentrations obtaining a sigmoid curve; IC50 was the inhibitor concentration needed to observe a 50% inhibition of the ACE activity and is expressed as mean value ± standard deviation of three independent assays.

In vitro DPP-IV inhibitory activity assay.

The experiments were carried out in a half volume 96 well solid plate (white). A total of 50.0 µL of each reaction was prepared in a microcentrifuge tube adding 30.0 µL of 1 × assay buffer [20 mM Tris-HCl, pH 8.0, containing 100 mM NaCl, and 1 mM EDTA], 10.0 µL of LTFPG (at the final concentration of 10, 100, and 500 µM), or vehicle (C, H2O) and 10.0 µL of purified human recombinant DPP-IV enzyme. Subsequently, reagents were transferred in each well of the plate and each reaction was started by adding 50.0 µL of substrate solution (5 mM H-Gly-Pro-AMC) and incubated at 37 °C for 30 min. Fluorescence signals were measured using the Synergy H1 fluorescent plate reader from Biotek (exc/em wavelengths 360/465 nm).

Targeted HPLC-Chip-MS/MS analysis: method set-up and validation

LC-MS/MS operating conditions.

The medium collected at the end of the experiments from AP and BL chambers (500 µL and 700 µL, respectively) were freeze-dried and residues were solubilized in HPLC water (100 µL). Samples were

desalted with C18 resin ZipTip (Millipore Corporation, Bedford, MA, USA). Each sample was lyophilized under vacuum and re-dissolved in 50 µL (0.1% formic acid), before the MS analysis. Purified BL samples were analyzed on a SL IT mass spectrometer interfaced with a HPLC- Chip Cube source (Agilent Technologies, Palo Alto, CA, USA). Data were processed with MSD Trap control 4.2, and Data analysis 4.2 version (Agilent Technologies). The chromatographic separation was performed using a 1200 HPLC system equipped with a binary pump. The peptide enrichment was performed on a 160 nL enrichment column (Zorbax 300SB-C18, 5 µm pore size), followed by separation on a 150 mm × 75 µm analytical column packed (Zorbax300SB-C18, 5 µm pore size). The samples (3 µL), previously acidified with formic acid, were loaded onto the enrichment column at a flow rate 4 µL/min using isocratic 100% C solvent phase (99% water, 1% ACN and 0.1% formic acid). After clean-up, P3, P5 and P7 were eluted into the mass spectrometer at the constant flow rate of 300 nL/min. The LC solvent A was 95% water, 5% ACN, 0.1% formic acid; solvent B was 5% water, 95% ACN, 0.1% formic acid. The nano-pump gradient program was as follows: 5% solvent B (0 min), 70% solvent B (0–8 min), and back to 5% in 2 min. The drying gas temperature was 300 °C, flow rate 3 L/min (nitrogen). Data acquisition occurred in positive ionization mode. Capillary voltage was −1950 V, with endplate offset −500V. Mass spectra were acquired in the mass range from m/z 50–2000, with target mass 700 m/z, average of 2 spectra, ICC target 30,000, and maximum accumulation time 150 ms. The LC/MS analysis were performed in MRM mode.

Calibration curve and quantification of P7.

Quantitative analysis of **P7** in AP and BL samples were carried out by the Ion Trap MS in MRM mode, monitoring two of the most intense diagnostic transitions for each peptide, after optimization of the acquisition parameters, such as retention time, MS profile, and MS/MS fragmentation spectrum. A blank was analyzed between samples to ensure absence of any carryover effects. Standard peptide solutions for the establishment of calibration curve were prepared at concentrations from 0,4 and 10 µM and analyzed by MRM to calculate peak areas. Then, AP and BL samples were

analyzed using the same optimized parameters. Data were processed with analysis Data analysis 4.2 version (Agilent Technologies).

Method validation

The analytical method was validated in terms of selectivity, linearity, limit of detection (LOD), limit of quantification (LOQ), accuracy and precision, according to the guidelines for bioanalytical method validation of the Center for Drug Evaluation and Research of the U.S. Food and Drug Administration (Food and Drug Administration 2001). Quality control samples were prepared by spiking each peptide in a BL sample from control Caco-2 cells at the concentrations of 0.5 μM.

Untargeted HPLC-Chip-MS/MS analysis for P7 metabolism analysis.

The metabolic degradation products assumed by the hydrolytic activity of brush border membrane peptidases were investigated through an untargeted approach. The equipment was the same above-mentioned, with the exception of the analysis conditions indicated below. Peptides were eluted according to followed gradient elution: 0–12 min, linear from 5 to 70 % B; 12–14 min, linear from 70 to 5% B; at a flow rate of 300 nL/min. The column outlet was directly coupled to an ESI, and the ion trap (MS/MS) was operated in positive polarity to acquire full scan mass spectra from m/z 300 to 2000. MS parameters for the specific peptide were nitrogen gas temperature, 300 °C; gas flow, 3.5 L/min; capillary, 2300 V; fragmentor, 85 V. Mass spectrometric data were processed using a Spectrum Mill MS Proteomics Workbench (Rev B.04.00, Agilent). The extraction of MS/MS spectra was conducted accepting a minimum sequence length of 3 amino acids and merging scans with same precursor within a mass window of ±0.4 m/z in a time frame of ±5 s. Methionine oxidation, acetylation (K), pyroglutamic acid (N-termQ), deamidated (N) were set as variable modifications, no enzyme was chosen as digestive enzymes; 2 missed cleavage were allowed. MS/MS search was conducted against the subset of Lupinus protein sequences (8669 entries) downloaded from UNIProtKB (http://www.uniprot.org/). The mass tolerance of parent and fragments of MS/MS data

search was set at 1.0 Da for precursor ions and 0.8 for fragment ions, respectively. Autovalidation strategy both in peptide mode and in protein polishing mode was performed using FDR cut-off ≤ 1.2 %.

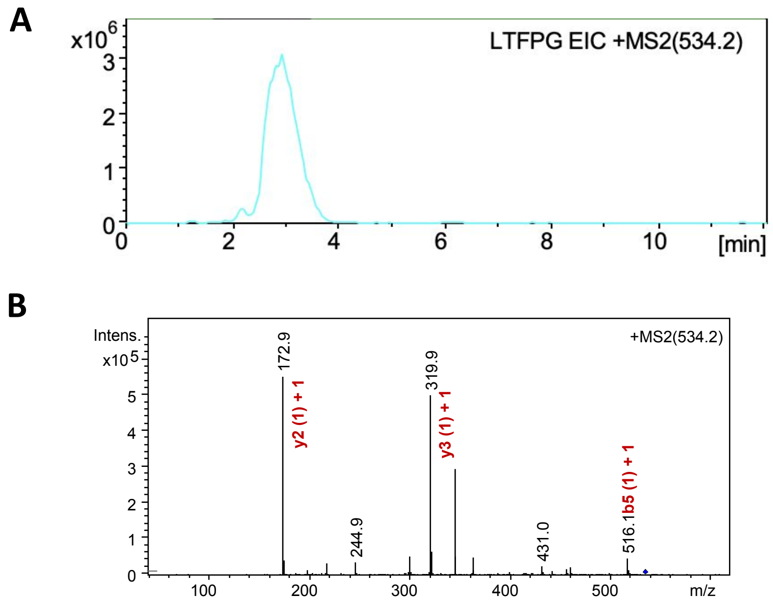

Figure 1S **(A)** EIC chromatogram of LTFPG and **(B)** tandem MS/MS spectrum of LTFPG.

Table 1S. In vitro ACE inhibition of P7

Peptide Sequence	ACE inhibition at 1mg/mL
LTFPGSDAD (P7)	10.9±0.95%
